# Virtual Screening for Potential Inhibitors of Human Hexokinase II for the Development of Anti-Dengue Therapeutics

**DOI:** 10.3390/biotech10010001

**Published:** 2020-12-28

**Authors:** Suriyea Tanbin, Fazia Adyani Ahmad Fuad, Azzmer Azzar Abdul Hamid

**Affiliations:** 1 Department of Biotechnology Engineering, Faculty of Engineering, International Islamic University Malaysia, Kuala Lumpur 50728, Malaysia; tanbin.khu.bd@gmail.com; 2 Research Unit for Bioinformatics & Computational Biology (RUBIC), Kulliyyah of Science, International Islamic University Malaysia, Bandar Indera Mahkota, Kuantan 25200, Malaysia; azzmer@iium.edu.my

**Keywords:** human hexokinase II, glycolysis, ligand-based screening, structure-based screening, molecular dynamics simulation

## Abstract

Dengue fever, which is a disease caused by the dengue virus (DENV), is a major unsolved issue in many tropical and sub-tropical regions of the world. The absence of treatment that effectively prevent further viral propagation inside the human’s body resulted in a high number of deaths globally each year. Thus, novel anti-dengue therapies are required for effective treatment. Human hexokinase II (HKII), which is the first enzyme in the glycolytic pathway, is an important drug target due to its significant impact on viral replication and survival in host cells. In this study, 23.1 million compounds were computationally-screened against HKII using the Ultrafast Shape Recognition with a CREDO Atom Types (USRCAT) algorithm. In total, 300 compounds with the highest similarity scores relative to three reference molecules, known as Alpha-D-glucose (GLC), Beta-D-glucose-6-phosphate (BG6), and 2-deoxyglucose (2DG), were aligned. Of these 300 compounds, 165 were chosen for further structure-based screening, based on their similarity scores, ADME analysis, the Lipinski’s Rule of Five, and virtual toxicity test results. The selected analogues were subsequently docked against each domain of the HKII structure (PDB ID: 2NZT) using AutoDock Vina programme. The three top-ranked compounds for each query were then selected from the docking results based on their binding energy, the number of hydrogen bonds formed, and the specific catalytic residues. The best docking results for each analogue were observed for the C-terminus of Chain B. The top-ranked analogues of GLC, compound 10, compound 26, and compound 58, showed predicted binding energies of −7.2, −7.0, and −6.10 kcal/mol and 7, 5, and 2 hydrogen bonds, respectively. The analogues of BG6, compound 30, compound 36, and compound 38, showed predicted binding energies of −7.8, −7.4, and −7.0 kcal/mol and 11, 9, and 5 hydrogen bonds, while the top three analogues of 2DG, known as compound 1, compound 4, and compound 31, showed predicted binding energies of −6.8, −6.3, and −6.3 kcal/mol and 4, 3, and 1 hydrogen bonds, sequentially. The highest-ranked compounds in the docking analysis were then selected for molecular dynamics simulation, where compound 10, compound 30, and compound 1, which are the analogues of GLC, BG6, and 2DG, have shown strong protein-ligand stability with an RMSD value of ±5.0 A° with a 5 H bond, ±4.0 A° with an 8 H bond, and ±0.5 A° with a 2 H bond, respectively, compared to the reference molecules throughout the 20 ns simulation time. Therefore, by using the computational studies, we proposed novel compounds, which may act as potential drugs against DENV by inhibiting HKII’s activity.

## 1. Introduction

Dengue is a major unsolved problem, complicated by factors including sequential infection with different dengue serotypes, increased populations, unplanned urbanization, and uncontrolled population increase of the leading vector, *Aedes aegypti.* The consequences of these factors are a resurgence in dengue epidemics and the introduction of dengue hemorrhagic fever and dengue shock syndrome to previously unaffected regions [1,2,3]. 

The clinical manifestation of dengue has a broad range of symptoms, from a minor influenza-like pattern, which is associated with high fever, vomiting, loss of appetite, severe headache, myalgia, and arthralgia, to a severe life-threatening illness characterized by plasma leakage, thrombocytopenia, hemorrhage (dengue hemorrhagic fever), and the potential to develop shock [4,5]. Global incidence of dengue has increased dramatically in recent years, with 390 million infections occurring annually. Of these cases, 96 million cases manifest clinically and 3.9 billion are at risk of dengue infection [6]. More than 70% of cases occur in the South-East Asia and Western Pacific regions [6]. According to the Pan American Health Organization (PAHO), approximately 3 million dengue cases are currently being reported in America alone, including 68 fatal cases. This represents a 42% increase compared to the previous year [7]. The incidence of dengue has also substantially increased in Asia. 

Dengue infection is caused by the dengue virus, which is a single-stranded RNA virus belonging to the Flaviridiae family. It is transmitted to humans from female mosquitoes of the *Aedes* genus, principally *A. aegypti,* through a single bite [6,8,9]. The positive-sense viral RNA, which has a length of approximately 11 kb, encodes three structural proteins (capsid, pre-membrane, and membrane) that help form the virion and seven non-structural proteins (NS1, NS2A, NS2B, NS3, NS4A, NS4B, and NS5) that aid in viral replication [10]. Dengue virus has four distinct antigenic serotypes: DENV-1, DENV-2, DENV-3, and DENV-4. Sequential infection with multiple serotypes of the virus can result in a more severe form of the disease [11,12]. 

Viruses are intercellular infectious agents that rely on the metabolic machinery of the host cell to survive and replicate. Several viruses, such as human cytomegalovirus (HCMV), herpes simplex virus 1 (HSV-1), hepatitis C virus, influenza A virus, human immunodeficiency virus type 1 (HIV-1), Kaposi’s sarcoma-associated herpesvirus (KSHV), and vaccinia virus (VACV) have been shown to cause extensive changes to the central carbon metabolic pathways of host cells during viral attack [13,14,15]. In recent years, Fontaine and colleagues have demonstrated how dengue virus can alter host metabolism by conducting intracellular metabolic profiling of mock-infected and dengue-infected primary human foreskin fibroblasts (HFFs) at different time points [15]. The experimental data suggested that central carbon metabolism, specifically the glycolytic pathway, is significantly disrupted during dengue virus infection. In dengue virus infected cells, glucose consumption was increased, as was expression of glucose transporter 1 (GLUT1) and human hexokinase II (HKII), which are important regulating enzymes in the glycolytic pathway [15]. In the presence of oxygen, normal cells metabolize glucose via glycolysis to produce ATP through oxidative phosphorylation within the mitochondria. In tumor and cancer-infected cells, glucose is metabolized in a different way from normal cells with production of ATP from aerobic glycolysis, in a phenomenon called the “Warburg effect.” In addition, normal cells require energy (ATP or GTP), which are also building block components to ensure specific cell functions, while tumor and cancer cells must replicate all their cellular components, thus, increasing their need for a biosynthetic building block (nucleotides, amino acids, and lipids). The main attribute of this metabolic changes is an increase in the glucose uptake by the cell. Extensive studies have postulated that virus-infected cells have the same Warburg-like phenomena where the key enzyme of glycolytic pathway, HKII contributes to enhanced glucose consumption in infected cells [16,17,18,19]. HKII is a rate-limiting enzyme of the glycolytic pathway that assist in increasing glucose consumption in the virus-infected cell. Hence, HKII has been chosen as a potential drug target. Fontaine and colleagues have also reported that glucose consumption tremendously increased during dengue viral infection, where glucose is metabolized via the host glycolytic pathway and provides energy (ATP) as well as metabolites to a dengue-infected cell. Consequently, virus takes this metabolic change as an advantage and assists themselves to survive and replicate inside the host cell. It has also been proven that HKII enhanced glucose consumption in the infected cell, as it is the key enzyme of the glycolytic pathway, which served as a basis for the enzyme to be a target in antiviral therapy [20]. Several studies on other viruses such as the poliovirus, HSV-1 [21], HCMV [22], HSV [23], and HCV [24] have also been documented, highlighting that glycolysis is crucial for the viruses to survive and replicate inside a host cell, where HKII governs the pathway.

Several experiments have shown that viral replication, as well as tumor cell proliferation, were positively correlated with glycolysis. This suggests that treatment with 2-deoxyglucose (2DG), which is a competitive antagonist of the glycolysis process, could inhibit viral replication [19,20]. 2DG is an analogue of glucose, in which two hydroxyl groups have been replaced by hydrogen atoms. This inhibits the production of glucose-6-phosphate, ultimately inhibiting the glycolytic pathway [25]. 2DG has been suggested by several studies for treating cancer, alongside inhibiting dengue virus replication [26,27,28]. However, 2DG has shown toxic effects when used to treat patients [19,29]. Hence, it is vital to find new compounds that are similar to 2DG, but do not induce toxicity, as these could be valuable candidates for anti-dengue drug development. Nevertheless, 2DG has been used as a competitive inhibitor of glucose for HKII in various viral therapeutic research, but, as previously shown, 2-DG has exhibited toxic effects on host cells [19]. Hence, the situation further urges finding potential inhibitors against HKII for the development of anti-dengue therapeutics. In addition to 2DG, both Alpha-D-glucose (GLC) and Beta-D-glucose-6-phosphate (BG6), which are the substrate and product of HKII, respectively, were used as reference molecules for rational drug design. 

In this study, we report the identification of small molecules, which have the potentials to be developed into safe and effective anti-dengue drugs. We first conducted a ligand-based drug design experiment by screening compounds similar to those described above. Subsequently, a series of docking and scoring experiments were conducted, which were followed by structure-based screening using the crystal structure of human HKII (PDB ID: 2NZT). These compounds were then subjected to virtual toxicity testing. Finally, the best-performing compounds were selected for Molecular Dynamics (MD) simulation analysis.

## 2. Materials and Methods

### 2.1. Ligand-Based Screening

#### 2.1.1. Identification of Lead Molecules and Analogues

In this study, ligand-based screening has been performed to filter drug-like compounds based on its query molecules with shape similarity, physiological properties, and toxicity from enormous compound libraries. Ligand-based screening provides a set of structurally-diverse ligands that bind to a receptor, where the approach of this screening is based on searching for molecules with a shape similarity to that of known actives, as such molecules will fit the target’s binding site [30]. Three different lead molecules Alpha-D-glucose (GLC), Beta-D-glucose-6-phosphate (BG6), and 2-Deoxyglucose (2DG) have been used as query molecules for initial pharmacophore screening. The common structural features of GLC, BG6, and 2DG have a glucose chain with six carbon atoms. Moreover, the functional feature of these query molecules (GLC, BG6, and 2DG) is possessing inhibitory effect to human HKII protein. It has been proven that, at a high concentration (up to 5 µM), GLC shows an inhibitory effect on HKII, while BG6 itself is an inhibitory product of HKII, while 2DG is a well-known inhibitor of HKII [17,29,31].

The structure data file (SDF) of GLC, BG6, and 2DG (with PubChem ID CHEBI: 10822 ID CHEBI: 17925 and ID CHEBI: 17719, as well as molecular formula C6H12O5 C6H12O6 and C6H13O9P) were extracted from PubChem (https://pubchem.ncbi.nlm.nih.gov/) to screen for the analogues by using Ultrafast Shape Recognition with CREDO Atom Types (USRCAT) available at http://usr.marseille.inserm.fr/. A total of 300 compounds, where, for each query of molecules, 100 similar hits have been countenanced from the USRCAT program. All predicted molecules ranked based on score zero (0) to one (1) score, where a compound with a score near to one indicated greater similarity toward its respective query molecule. Furthermore, a total 165 hits out of 300 has been screened based on Absorption, Distribution, Metabolism and Excretion (ADME) analysis, a toxicity test, and a Lipinski’s Rule of Five. Later, selected compounds were screened through a structure-based approach and, finally, were analysed through molecular dynamics simulation studies.

#### 2.1.2. ADME Analysis

Physiochemical properties such as absorption, distribution, metabolism, and excretion for all compounds were predicted from the SwissADME website (www.http://www.swissadme.ch/index.php), prior to a docking study. Structure data file (SDF) for each compound was used to obtain ADME values. All selected compounds fulfilled the benchmarks of ADME analysis.

#### 2.1.3. Toxicity Test

In a drug design process, the prediction of compound toxicity is crucial to identify non-toxic compounds. In this experiment, we used ProTox-II (www.http://tox.charite.de/protox_II/) to determine the non-toxicity of selected compounds. The SDF format of each compound was converted into a molecular data file (MDL Molfile) using Open Bable software. Various toxicity end points such as acute toxicity, hepatotoxicity, cytotoxicity, mutagenicity, and immunotoxicity have been tested to predict the level of toxicity of each compound. In ProTox-II, toxic levels were classified in six different forms, Class-I to Class-VI, based on a range of lethal dose (LD50) values, where Class-I determines the toxic compound, while Class-VI determines a non-toxic compound.

### 2.2. Structure-Based Screening

#### 2.2.1. Retrieval and Structural Preparation of the Protein

HKII is a homodimer protein with highly similar N and C terminal domains that together form half of the protein. The crystal structure of HKII (PDB ID: 2NZT) was retrieved from the Protein Data Bank website (https://www.rcsb.org/). The native structure of the protein was complexed with a heteroatom, a water molecule, and an unknown unique ligand (UNX), which are crucial to be removed to ensure good protein-ligand interaction. The water molecule might deform the binding site residues as well as the interactions between protein-ligand atoms. On the other hand, the heteroatom and unknown ligand have to be removed due to docking of the selected ligand and to facilitate a smooth molecular docking process. Structural visualization was conducted using PyMOL software.

#### 2.2.2. Molecular Docking

Molecular docking is usually referred to as protein-ligand binding. The aim of docking is to understand molecular recognition, a favourable binding pose, and binding affinity of ligand toward the 3D structure of the receptor protein. The protein structure was prepared by protein optimization and energy minimization by adding hydrogen atoms, as well as removing an existing ligand from the active site. The known ligands (GLC and BG6) were removed for a re-docking purpose, where the active site of the protein structure is typically being validated by re-docking experiments [32,33,34]. The ligands (GLC and BG6) were extracted from native protein-ligand complexes to evaluate the conformation and, subsequently, docked back into the protein to validate the docking location. In this case, we scrutinized the native structure of HKII using PyMOL and observed that there is a cleft inside both N-terminal and C-terminal domain in the protein, where two known ligands (BG6 and GLC) were located in parallel. The selected compounds and their query molecules were docked inside the cleft sequentially. It should be noted that only the N-terminal domain was used to conduct the docking process because the N-terminal domain regulates the stability of the whole enzyme, in contrast with the C-terminal domain [35,36]. Recently, Nawaz and colleagues have reported that the human hexokinase II is overexpressed in tumours and predominantly found on the outer mitochondrial membrane where its N-terminus initiates and maintains the tumorigenesis. They further crystalized and generated the protein’s structure using MODELLER [36,37]. However, the catalytic residue of human hexokinase II has not yet been publicly available. Hence, it was a challenge to find the active site residues of HKII. In this experiment, we scrutinized the catalytic residues around the cleft of HKII, where the known ligands (GLC and BG6) were bound using MGLTools (version 1.5.7), which were further validated by re-docking processes. In this research, we used the AutoDock Vina program that significantly improved the average accuracy of the binding mode predictions. In the Vina script, all information must be included such as grid box size (Box size: 26 ×24 × 22 A°), grid box centre (X = 43.567, Y = 35.439, Z = 36.27), and measurement of space (1.000), where the defined grid box encompasses the protein binding site to do sequential docking of all selected ligands. The ligands were prepared by adding a non-polar hydrogen atom using Avogadro. Since AutoDock Vina can only accept a Protein Data bank, Partial Charge (Q), and atom Type (T) format (PDBQT), the PDBQT format of the HKII protein, and all grid box information were occupied using MGLTools, while the SDF files of all ligands were converted to a PDBQT file using OpenBable software.

### 2.3. Molecular Dynamics Simulation

**Figure 1:**
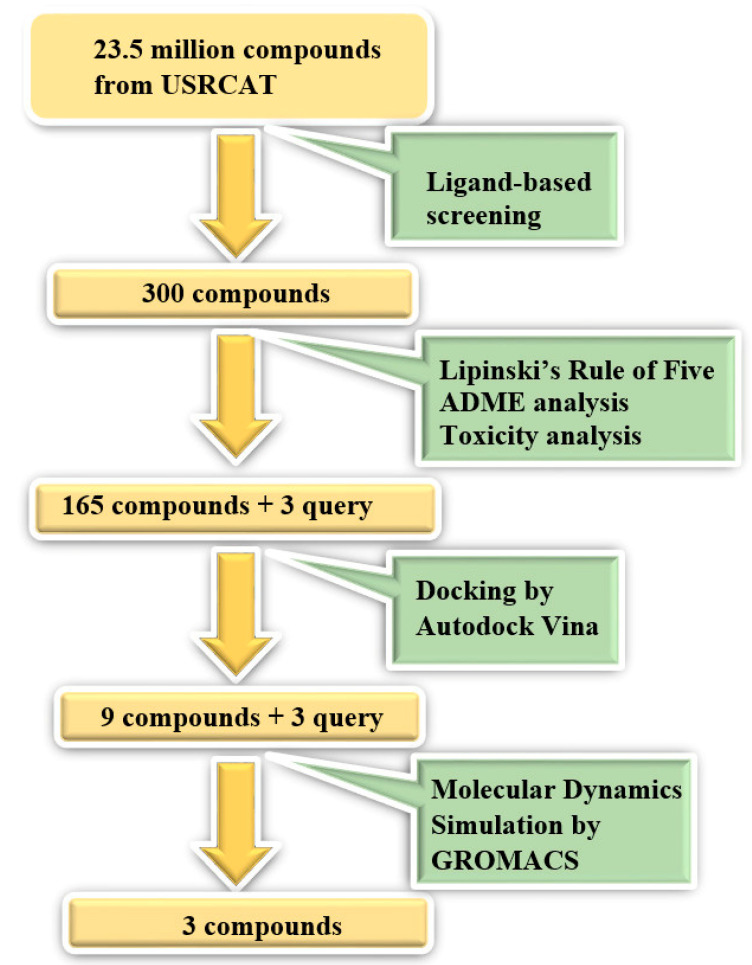
The virtual screening work flow. The process begins with ligand-based screening, selection of drug-like molecules by Lipinski’s Rule of Five, ADME analysis, toxicity analysis, docking, and Molecular Dynamics simulation.

Molecular dynamics simulation has been widely used to scrutinize the behaviour of molecules at an atomic level, in order to understand the structure and interactions between a protein and a ligand molecule in a period of time. We set up molecular dynamics simulation of selected compounds against HKII by using GROMACS version 5, where the .pdb format of protein and selected compounds from molecular docking were converted to a GROMACS file format to be implemented in the MD simulation. Atomic interactions were calculated with a gromos96 54a7 force field and all systems were solvating in a cubic box with a simple point charge (SPC) pre-equilibrate water system. Ligand topology files were extracted from the Automated Topology Builder (ATB) (https://atb.uq.edu.au) server since GROMACS does not generate a ligand topology file. We used standard cut-off 1.0 nm for short-range coulomb interaction, where statistical and energy data are stored for every 10 steps. The v-rescale and Berendsen weak coupling algorithm were used to control temperature and pressure, respectively, during the simulation process. The water equilibration around the protein-ligand complex is crucial to avoid unnecessary exaggeration of the complex when the MD simulation has started. In order to avoid this problem, we performed an equilibration run for 100 ps, where all heavy atoms were restrained at their start position. Finally, 20 ns constrained MD simulation was performed for each selected protein-ligand complex. All bond lengths, including the hydrogen atoms were constrained by the LINCS algorithm. After simulation has completed, we viewed the trajectory by using VMD and analysis such as RMSD, distance between protein and ligand throughout the simulation, and formation of the hydrogen bond, which were carried out using Xmgrace software. The virtual screening workflow is presented in Figure 1.

## 3. Results and Discussion

### 3.1. Ligand-Based Screening

#### 3.1.1. Identification of Analogues

In pharmaceutical research, *in silico* screening has become prominent day-by-day. It is a computer-based method divided into ligand-based screening and structure-based screening. The former is the initial stage of virtual screening, which is the most exoteric approach for drug discovery and lead optimisation. The spotlight of this technique is to compare molecular similarity with known and unknown fractions [32,33]. Ligand-based screening exploits key structural and physiochemical properties of ligands and target to enable the screening of active compounds, while structure-based screening most widely used for molecular docking by exploiting the three-dimensional (3D) structure of the target protein and predicting the preferred protein-ligand binding pose through the scoring function [30,33,38]. In this work, USRCAT (Ultrafast Shape Recognition with CREDO Atom Type) program has been used to screen similar compounds of Alpha-D-glucose (GLC), Beta-D-glucose-6-phosphate (BG6), and 2-deoxyglucose (2DG) against HKII, where GLC and BG6 are the substrate and product of HKII, respectively, while 2DG is a known inhibitor of HKII. USRCAT is a publicly-accessible web tool, which is able to screen 50 million conformers per second. USRCAT is an extended version of USR (Ultra shape recognition) algorithm, which is improved with the CREDO database, where users are able to find an enormous chemical component ranging from a natural product to drug-like molecules as well as solvent molecules and ions. USRCAT is an advanced version of UFSRAT in terms of having high capability to differentiate similar molecules at an atomic level and intended to discriminate between long chain-like molecules, such as heteropeptides and long alkayl chains [39]. The USRCAT algorithm is based not only on shape similarity but also considers geometrical distribution of atoms such as all atoms, a hydrogen bond donor, and acceptor atoms, as well as aromatic atoms [39].

In this study, 23.1 million molecules were screened and a total of 300 most similar compounds were obtained from USRCAT for all of the reference molecules (2DG, GLC, and BG6). Thus, specifically, each query has 100 similar compounds aligned into the hits.csv file along with similarity scores, physicochemical properties, and the vendor’s information. Similarity was evaluated based on the scoring function ranging from 0–1, where zero and one indicates minimum and maximum similarity, respectively. As mentioned above, the USRCAT score zero indicates the least similar compounds, while those closer to one reflects a much closer resemblance toward the reference molecule. Based on these findings, a total of 165 compounds out of 300 similar hits for each query molecule were chosen and their physiological properties were obtained from the ZINC database. The ZINC database contains 727,842 purchasable compounds from various suppliers (ChemBridge, ChemDiv, Ryan, Asinex, Sigma-Aldrich, Maybridge, Specs, Comgenex, and Otava) in which 494,915 are Lipinski subjacents. Based on the similarity scores, the three top-ranked compounds that resembled each of the query molecule are shown in Table 1, Table 2 and Table 3, as their ranking scores ranged from 0.946 to 0.913, 0.817 to 0.812, and 0.806 to 0.751 for GLC, BG6, and 2DG, respectively. All three compounds, which are analogues of 2DG, GLC, and BG6, which have been selected for the subsequent studies, obeyed the Lipinski’s Rule of Five in terms of their physiological parameters, hydrogen bond donors ˂5, hydrogen bond acceptor ˂10, molecular weight under ˂500 g/mol, and a partition coefficient logP ˂ 5 with no violation [40]. The mentioned parameters related to the Lipinski’s Rule of Five are presented in Table 1, Table 2 and Table 3. Compounds 10 to 58, which are similar conformers of GLC possess MlogP values ranging from −2.15 to −1.93, and drug-likeness from 0.14 to −0.01 (Table 1). Moreover, the top three similar hits of BG6, compounds 30 to 38 have MlogP values from −4.02 to −3.84 and drug-likeness from 0.89 to −1.08 (Table 2). Drug-likeness is the preliminary concept of drug design and its value is usually estimated from the compound’s molecular structure standardized between −0.4 and 5.6, whereby it indicates whether the compound has drug properties or not. Since our findings were optimized with respect to the drug-likeness parameter and the adherence to the Lipinski’s Rule of Five, most of the compounds fulfilled all the criteria. The drug-like properties of 2DG corresponded to the MlogP values between 0.06 and −0.08, and drug-likeness between −0.64 and −1.16 (Table 3). It is noteworthy to note that, although the same scores were obtained from the USRCAT program, these compounds are structurally different and contain different atom types. The Rule of Five provides insights for the analysis of the molecular properties and structural features of every compound, which is important for drug pharmacokinetics in a human’s body, including their absorption, distribution, metabolism, and excretion (ADME) [41].

Computational methods are effectively used in pharmaceutical research to improve the drug discovery process. Ligand-based screening is one of the crucial parts of *in silico* drug design and several novel compounds have been invented through this process. Hence, considering the importance of a computational drug design method, it was adopted in the present study to discover the potential HKII inhibitors. Numerous successful ligand-based screening investigations have been performed, aiming to identify potential inhibitors against dengue drug targets. In extant studies, dengue virus encoded non-structural protein 3(NS3) was used as a drug target, and two known inhibitors, namely suramin and ivermectin, have been successfully identified through virtual screening and *in vitro* analysis, where researchers used a LOPAC compound library for inhibitor selection as well as vero-B and vero-E6 cell for an in vitro assay [42,43]. It is noteworthy to note that most of the research conducted to search for anti-dengue drugs have been conducted against viral replication proteins or viral envelop proteins, but our research focus is on a human protein as a drug target. There are numerous web-based programs available to implement this process, whereas, in this work, an online program, USRCAT, has been utilized, which was incorporated with a ZINC database. Lipinski’s Rule of Five functions as a filter to choose drug-like compounds based on their molecular property, including absorption, distribution, metabolism, and excretion. Selected compounds were chosen based on the similarity scores and drug-like criteria.

#### 3.1.2. Physiochemical Properties Analysis (ADME)

ADME studies are crucial parts of any drug development program and essential for obedience with regulatory guidelines [41]. In this work, an acceptable range in the values of predicted physiochemical and pharmaceutical properties of selected compounds has been achieved, which includes absorption, distribution, metabolism, and excretion (Table 4, Table 5 and Table 6). The water solubility of a compound reflects the solubility of the molecule in water at 25 °C, where the threshold of the solubility is ˂0.05 log mol/L. The lipid-soluble drug is less well-absorbed than water-soluble ones. The Caco-2 permeability cell line is composed of human epithelial colorectal cells, which is widely used as an *in vitro* model of the human intestinal mucosa to predict the absorption of orally-administered drugs [44].

The water solubility of a compound reflects the solubility of the molecule in water at 25 °C. A lipid-soluble drug is less well-absorbed than water-soluble ones. The Caco-2 permeability cell line is composed of human epithelial colorectal cells, which is widely used as an *in vitro* model of the human intestinal mucosa to predict the absorption of orally-administered drugs [44]. These models predicted the Caco-2 permeability values using a logarithm of the permeability coefficient (log cm/s) based on the 647 drug-like molecules. For the ADME predictive model, a high Caco-2 permeability value would be ˃0.90. Furthermore, intestinal absorption is a primary site for absorption of a drug orally, where a less than 30% predated value is considered poor absorption while logKp ˃ –2.5 defines the low skin permeability. This predictive model was built using the measured free proportion of 552 compounds in human blood (Fu), where the predicted value should be less than 2.81 log10^−6^ cm/s and logBB ˃ 0.3 as well as logPS ˃ 2 was considered to readily cross the blood brain barrier (BBB) and penetrate the central nervous system, respectively [41,44]. However, our findings successfully met the parameter of absorption for each query molecule and the selected analogues.

The results (Table 4, Table 5 and Table 6) revealed that all compounds possess good water solubility, Caco2 permeability, human intestinal absorption, and skin permeability values. Greater values of these parameters denote that the compound could be better absorbed from the intestinal tract upon oral administration. Moreover, fraction unbound values (Fu ˂ 1) indicate that the compounds exist in plasma at an unbound state and could efficiently travel through the cellular membrane. In addition, all compounds displayed negative penetration through the Blood Brain Barrier (BBB) and Central Nervous System (CNS), which means that these compounds do not cross the BBB and CNS (Table 4, Table 5 and Table 6).

CYP2D6 and CYP3A4 are two isoforms of Cytochrome p450, which is a crucial detoxification enzyme of the human body and responsible for changing the pharmacokinetics of drugs. Thus, the tested compounds should be inactive (Table 4, Table 5 and Table 6). In terms of metabolism, we found that all compounds act as a non-inhibitor of the cytochrome p450, which means that the molecule will not restrict the biotransformation of the drug metabolized by the CY2D6 enzyme and the CYP1A2 enzyme. Furthermore, renal OCT2 is an organic cation transporter 2, which plays a crucial role in renal clearance and disposition of drugs. Thus, tested compounds should not act as inhibitors of OCT2 and has to be inactive during experiments. 

In excretion, we found that all compounds are inactive for renal OCT2 inhibitors, while the predicted LD_50_ value of these compounds are ˃500 mg/kg, suggesting that these compounds do not possess acute oral toxicity at lower doses (Table 4, Table 5 and Table 6). Important information obtained from SwissADME was the computed LD_50_ dose in a rat model. By comparing the LD_50_ doses, a compound with a lower dose was found to be more lethal than the compound with a higher LD_50._ From our observation, all compounds possess higher LD_50_ values (Table 4, Table 5 and Table 6). The physiochemical properties showed that all compounds, which are analogues of GLC, BG6, and 2DG acquiring drug-like properties, can be good inhibitors of human HKII protein when tested *in vitro*.

#### 3.1.3. Toxicity Test

The toxicity test in this study was conducted by using the Pro Tox-II program. The prediction of compound toxicities is an important part of a drug design development process. Computational toxicity estimations are not only faster than the determination of toxic doses in animals, but may also help reduce the amount of animal experiments. ProTox-II incorporates molecular similarity, fragment propensities, most frequent features, and (fragment similarity-based CLUSTER cross-validation) machine-learning, based on a total of 33 models for the prediction of various toxicity endpoints such as acute toxicity, hepatotoxicity, cytotoxicity, carcinogenicity, mutagenicity, immunotoxicity, adverse outcomes (Tox21) pathways and toxicity targets [45]. Acceptable toxicity value of GLC, BG6, and 2DG analogues have been tabulated in Table 7, Table 8 and Table 9, respectively. The toxicity has been classified into five classes: Class I: fatal if swallowed (LD50 ≤ 5), Class II: fatal if swallowed (5 < LD50 ≤ 50), Class III: toxic if swallowed (50 < LD50 ≤ 300), Class IV: harmful if swallowed (300 < LD50 ≤ 2000), Class V: may be harmful if swallowed (2000 < LD50 ≤ 5000), and Class VI: non-toxic (LD50 > 5000). Toxicity is proven essential in examining compounds for potential inclusion into drug development. The aim of toxicity studies is to ensure safety of the chemical compounds before they can be used as drugs or during clinical trials, as well as to determine the toxic effects of test substances [46]. In several experiments, different types of toxicity tests (acute toxicity, sub-acute toxicity, and chronic toxicity studies) have been conducted to characterize the possible toxic effects of drugs that can range from minor to critical [46,47].

The Pro-tox-II software utilizes the consensus method to determine the values of different compounds by using different tests end-point analysis approaches, where LD50 and LC50 values are exhibited for each compound (Table 7, Table 8 and Table 9). A smaller value indicates a more toxic compound and vice versa. According to the data analysis results obtained in the present study, most of the compounds that were similar to 2DG were toxic, but, in contrast, the toxicity of the top-ranked analogues of GLC exhibited non-toxic effects. Moreover, the analogues of BG6 were predicted to be non-toxic. It should also be noted that all examined compounds were negative for mutagenicity. As already mentioned previously, 2DG is a known inhibitor of HKII, but it has shown a toxic effect in in *in vitro* analysis [19,20], which highlighted the urgency to search for compounds that can be developed into a potent HKII inhibitor, which are non-toxic and safe to be administered as medications.

### 3.2. Structure-Based Screening

Structure-based virtual screening (SBVS) is an impactful experiment instrumental for fast and cost-effective lead discovery and optimization. The aim of this process is to understand the three-dimensional structure of the biological target at a molecular level [48]. Structure-based screening states the interaction of protein-ligand binding, where the mode of the binding complex can be predicted through docking and scoring functions, that comprise a two-step calculation to calculate the free binding energy of complex and exploring the conformational space of a protein and ligand complex [48]. The advantages of SBVS are time-saving and cost effective. There is no need for physical existences of the molecule and there are available free access software and web tools for conducting SBVS [48,49]. There are some disadvantages too. SBVS can generate a false positive and a false negatives result, as some tools work in specific cases and not in general, while it is also difficult to predict the accurate binding positions [49].

In this experiment, the crystal structure of human HKII from the Protein Data Bank (PDB ID: 2NZT) was prepared for molecular docking analysis by protein optimization and energy minimization. Additionally, all heteroatoms and ligands, which were already in a complex with the protein, were removed, because they can affect the docking process. HKII consists of two protein chains with two domains each. All 12 compounds were docked sequentially in each terminal of both chains, even though the two chains are homodimers. This was done to observe variations in the binding energy and hydrogen bond numbers between the two chains, allowing us to determine which of the two was the best for the subsequent simulation procedure. In this study, we have conducted a docking procedure on the N-terminal of chain A because several studies have suggested that the N-terminus (residues 1-475) contains more catalytic sites [35]. Our analysis involved docking 12 compounds with the selected catalytic residues for each of the query molecules (2DG, GLC, and BG6). All the top-ranked molecules were successfully docked in the catalytic site of human HKII.

**Figure 2:**
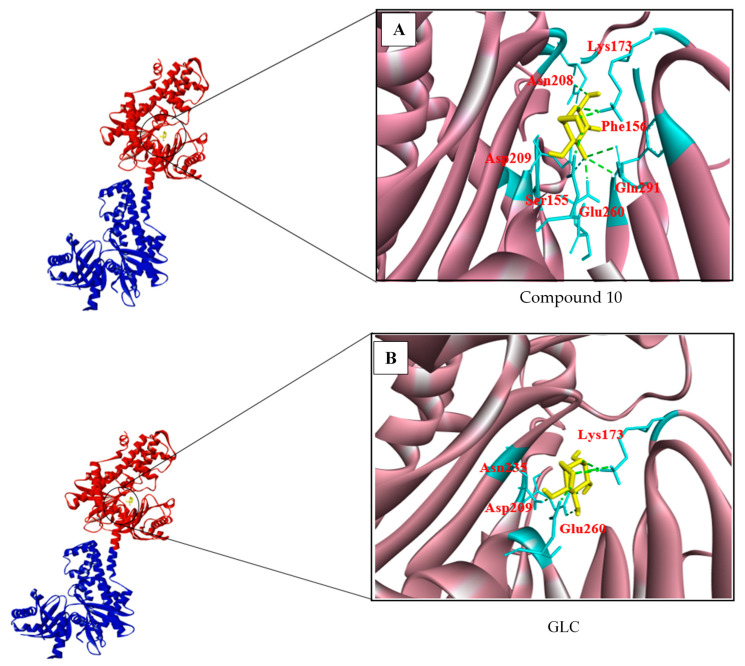
The chain A, N-terminal (red colour) and C-terminal (blue colour) of HKII was indicated in ribbon representation and the black circle indicates the catalytic site, where GLC and its analogues were docked. (**A**) Compound 10, which has formed one H bond with Gln291, Asp209, Phe156, and two H bond formed with Lys173, Asn208, and Glu260. (**B**) Meanwhile, the catalytic residue Lys173 has shown three H bonds, Asp209 with one, Asn235 with one, and Glu260 with one H bond, each formed with GLC.

The top-ranked analogues of GLC, known as compound 10, compound 26, and compound 58, had binding energies of −7.2, −7.0, and −6.10 kcal/mol, respectively, whereas the binding energy of GLC itself was −7.2 kcal/mol on the N-terminus of Chain A (Table 10). As described previously, the binding energy required for either chain of the HKII homodimer was similar. Among all analogues of GLC, compound 10 exhibited the best binding energy (−7.0 kcal/mol) and took part in seven hydrogen bonds with residues Lys173, Asn208, Asp209, Glu260, Phe156, and Gln291 on HKII (Figure 2A). This closely resembles the query molecule GLC, which has a binding energy of −7.2 kcal/mol and takes part in six hydrogen bonds with residues Glu260, Lys173, Asp209, and Asn235 on HKII (Figure 2B). However, compound 10 exhibited the best binding characteristics of the tested GLC analogues and might act as a potential inhibitor of HKII. 

**Figure 3:**
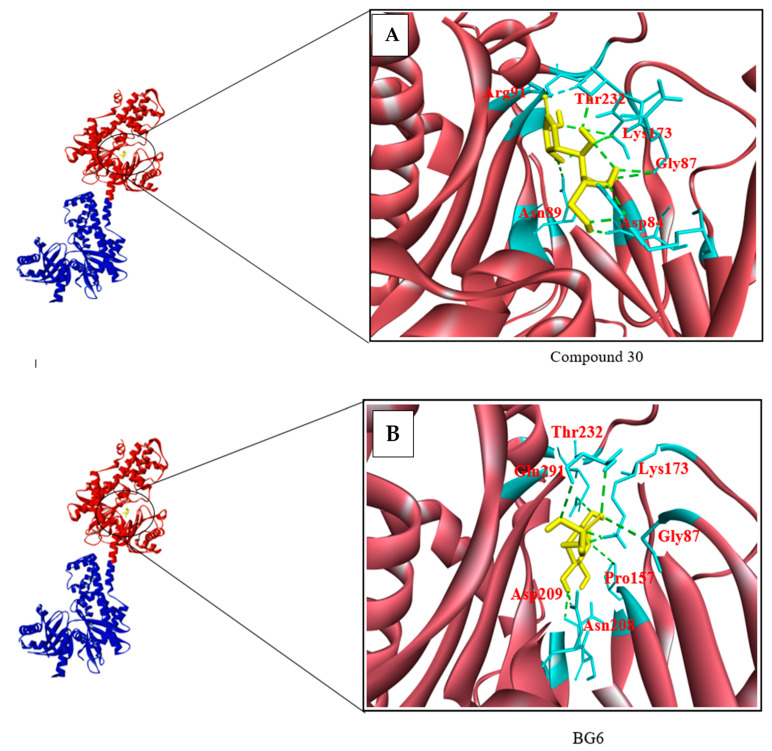
The chain A and N-terminal (red colour) and C-terminal (Blue colour) of HKII was indicated in a ribbon representation and a black circle indicates the catalytic site, where BG6 and its analogues were docked. (**A**) Compound 30 has formed one H bond with a catalytic residue Asn89 and Arg91, while Thr232, Lys173, and Gly87 formed two H bonds and Asp formed three H bonds with compound 30. (**B**) Meanwhile, BG6 has formed one H bond with each residue Gln291, Asn209, Asp208, Thr233, and Lys173.

The analogues of BG6, known as compound 30, compound 36, and compound 38, exhibited binding energies of −7.8, −7.4, and −7.0 kcal/mol, respectively, compared to BG6 itself, which has a binding energy of −7.9 kcal/mol (Table 11). Compound 30 was chosen as the analogue with the best binding characteristics because it had the lowest binding energy value (−7.8 kcal/mol) and forms eleven hydrogen bonds, with Gly87, Asn89, Thr232, Arg91, Asp84, and Lys173 (Figure 3A). BG6 forms up to six hydrogen bonds with Lys173, Asn208, Gln291, Asp209, Thr233, Pro157, and Gly87 (Figure 3B). Therefore, the compound with the best binding characteristics among BG6 analogues was compound 30, which may be a potential inhibitor of HKII. 

**Figure 4:**
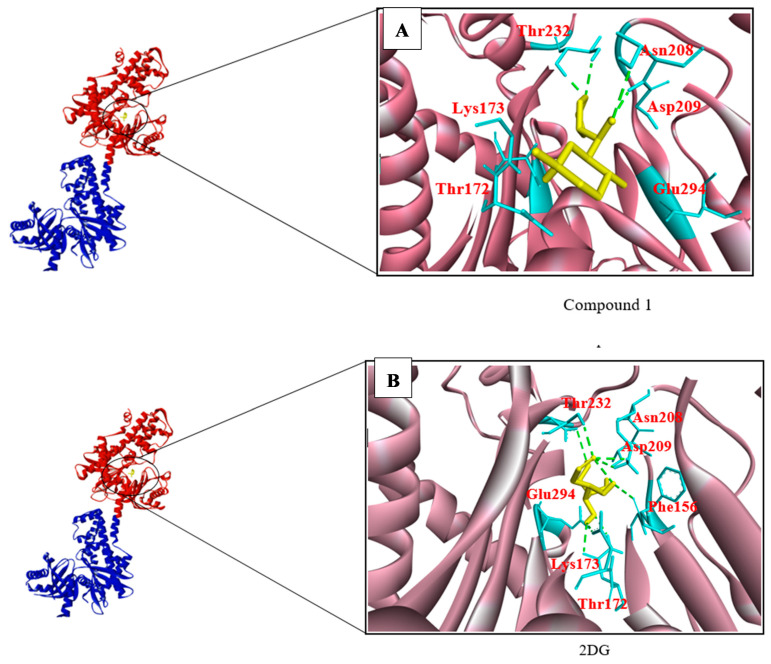
The chain A and N-terminal (red color) and C-terminal (Blue color) of HKII was indicated in a ribbon representation and the black circle indicates the catalytic site, where 2DG and its analogues were docked. (**A**) Compound 1 formed four H bonds with catalytic residues Thr232 and Asn208. (**B**) Meanwhile, 2DG has formed one H bond with each residue Glu294, Asn208, and Thr232, while two H bonds formed with Lys173, Asp209, and Phe156.

The 2DG analogues, known as compound 1, compound 4, and compound 31, exhibited binding energies of −6.8, −6.3, and −6.3 kcal/mol, respectively, as shown in Table 12. It should be noted that compound 1 exhibited a relatively low binding energy (−6.8 kcal/mol), despite forming four hydrogen bonds with Asn208, Asp209, Thr172, Glu294, Thr232, and Lys173 (Figure 4A), compared to the binding energy of 2DG (−6.0 kcal/mol), which forms eight hydrogen bonds with Asn208, Asp209, Thr172, Glu294, Lys173, Glu260, and Phe156 (Figure 4B). Therefore, compound 1 was selected for further inhibition analysis due to its relatively high binding affinity, compared to the other analogues of 2DG, and 2DG itself.

Previous studies have attempted to use molecular docking approaches to identify small molecule inhibitors of dengue virus proteins. For example, Lim and colleagues [50] attempted to identify NS5 methyltransferase (MTase) inhibitors using a molecular docking approach. NS5 is a non-structural viral protein located on the dengue virus surface that assists in viral replication. Hence, it has been proposed as a drug target, with ribavirin triphosphate (RTP) and S-adenosyl-L-methionine (SAM) acting as query molecules. Lim and colleagues reported that compounds SPH1-103799, SPH1-101-102, and 28SPH1-115-917 had the highest binding affinities of −11.4 kcal/mol, −11.4 kcal/mol, and −10.0 kcal/mol, respectively [50]. In this study, AutoDock Vina and EDULISS (Edinburgh University Ligand Selection System) were used for structure-based and ligand-based screening, respectively. Another study by Wang and colleagues used four-stage computational high-throughput screening to find a small molecule directed against the dengue virus envelope protein (E protein) [51]. The 23 highest ranking compounds in their analysis were selected based on the E protein binding site from three NIC libraries and were subsequently tested for antiviral activity in a biological assay. One compound, PO2, was found to inhibit viral reproduction. 

Thus, extensive research on the dengue virus has been conducted using molecular docking approaches [50,51,52,53,54,55,56,57]. However, it is important to note that all of the previously mentioned literature reported inhibitors which were developed based on viral proteins. There are plenty of *in silico* research on viral proteins were not fruitful for the development of dengue drug. Thus, in this research, we focused on human protein as a drug target. Overall, structure-based processes, such as molecular docking, provide a good approximation of protein-ligand binding affinity and help to identify the best candidates for further inhibition studies in antiviral drug development.

### 3.3. Molecular Dynamics Simulation

**Figure 5:**
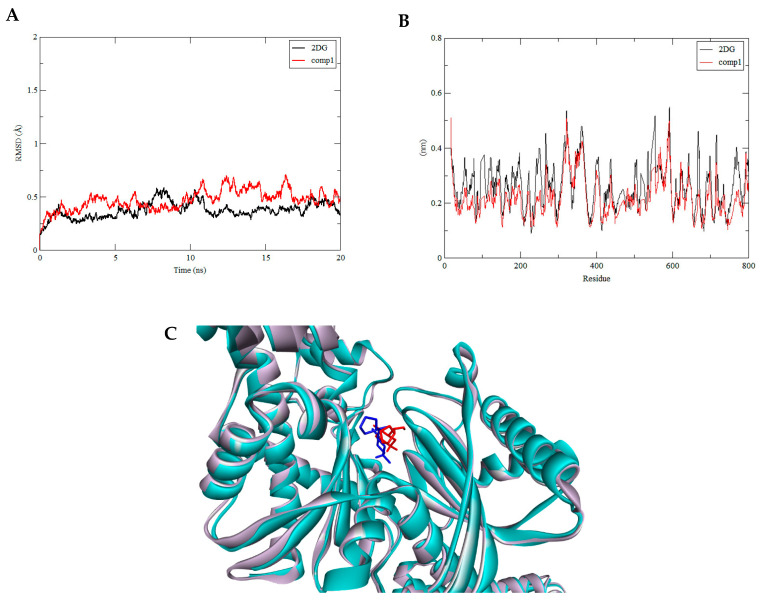
(**A**) The backbone RMSD of HKII-2DG (black) and HKII-compound 1(red) complexes shown during 20 ns of the MD simulation. (**B**) Residue RMSF of HKII-2DG and HKII-compound1 complexes generated during the trajectory period of 20 ns of the MD simulation. (**C**) Superimposition of the trajectory structure at 10 ns and 20 ns of MD simulations. The protein was presented in a cyan-coloured ribbon format. Snapshots of superimposed ligand 2DG (red) and compound 1(blue) located in a ligand binding site.

The structural deviations and stability of HKII docked with compound 1, compound 10, and compound 30, which are analogues of 2DG, GLC, and BG6, respectively, were assessed by 20 ns of MD simulation using GROMACS 4.6.5 programme. The stability of the protein-ligand complex was observed by measuring the root-mean-square deviation (RMSD) of each trajectory obtained in an MD simulation with respect to their position in the reference frame. The RMSD of all selected compounds fit into the HKII protein during the 20 ns period of dynamics simulation studies that are shown in Figure 5, Figure 7, and Figure 8. Ismail and Jusoh confirmed that RMSD determines the deviation in the average distance of the atomic structure movement in complexes, where generally the threshold of RMSD is 2Å [58]. The complex structure namely HKII-2DG, HKII-BG6, and HKII-GLC were used as references and comparison. Throughout the simulation, the complex HKII-Compound 1 and HKII-2DG were seen equilibrated and their protein backbones were in minimal deviations after 15 ns of simulation. In detail, the backbone of the complex HKII-compound 1 showed a slight fluctuation compared to the reference HKII-2DG complex toward the end of the simulation with the RMSD values consistent at ±0.5Å (Figure 5A), indicating that the interaction of HKII with compound 1 was comparable to 2DG. The HKII-2DG and HKII-compound 1 complexes were snapshots at one point of production simulation and both structures were superimposed to assess the ligand-binding topology as in Figure 5C. Both substrates were seen in a similar coordination, thus portraying the same level of inhibition towards the enzyme. The binding interaction of the HKII-compound 1 and HKII-2DG complexes were closely monitored and differentiated along the simulation trajectory. For root mean square fluctuation (RMSF), each amino acid fluctuation of HKII for compound 1 was in a similar pattern with reference compound 2DG (Figure 5B). No noteworthy changes were seen for the movement of the amino acids and the compounds did not disrupt the structural integrity of HKII.

**Figure 6:**
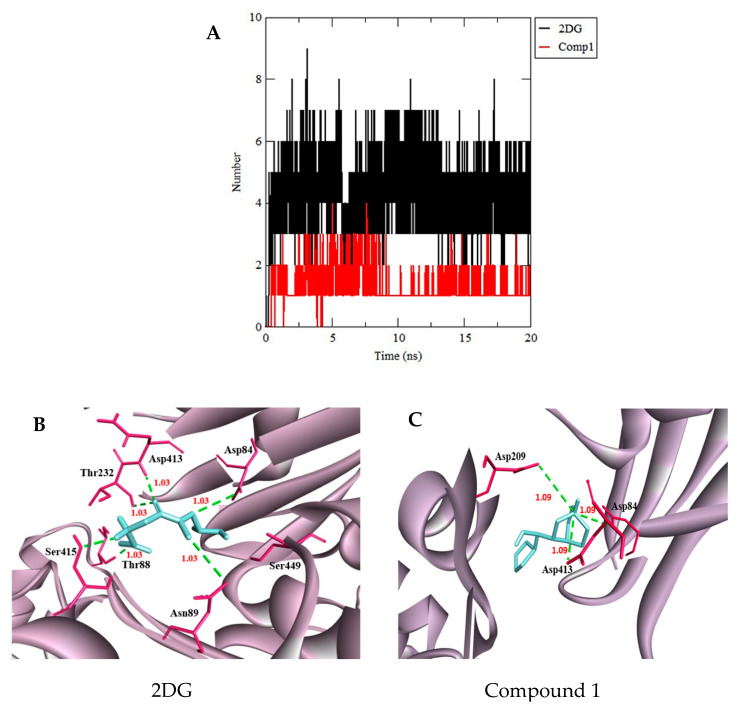
(**A**) Total number of hydrogen bond interactions between HKII and 2DG (black) and HKII and compound 1 (red). Snapshots of complex (**B**) HKII-2DG and (**C**) HKII-compound 1 were extracted from the trajectory at 20 ns. HKII-2DG has shown six H bonds. Two formed with Thr232 and Asp413, one formed with Asn89, one formed with Asp84, and two formed with Thr88, Ser415. The HKII-compound 1 has shown three H bonds. Three formed with Asp413, Asp84, and Asp209. The distance between HKII and 2DG (cyan), compound 1(cyan) are, on average, 1.03Å and 1.09Å, respectively.

The binding stabilities of the protein-compound complexes were monitored during the trajectory period of the MD simulations. The stabilities of the protein-compound complexes were evaluated by calculating the H bond profiles using the g, h bond tool of Gromacs [58,59]. The binding analysis revealed that the complex of 2DG in HKII had formed an average of six hydrogen bonds higher than the HKII-compound 1 complex (three hydrogen bonds) over the simulation time (Figure 6B,C). Figure 6B,C have shown the extracted trajectory of HKII-2DG and HKII-compound 1 at 20 ns, where compound 1 bind with HKII by three H bond with catalytic residue of Asp84, Asp209, and Asp413, while the reference compound 2DG showed six H bonds with residue Asp84, Asn89, Asp209, Thr232, Thr88, and Ser415 (Figure 6B,C). This analysis has suggested that 2DG has a strong interaction with HKII in the dynamic condition during MD simulation. However, the formation of H bonds for compound 1 are lower than 2DG, but the compound consistently bound in the active site of HKII stably projected the active site vicinity. Another useful analysis is a distance calculation between the HKII and selected compounds. Since our aim is to obtain the stability of protein-ligand binding, the distances between the ligand-protein have to be as close as possible. Figure 6B,C depict the distance between HKII and compound 1 and reference molecule 2DG, which were 1.09Å and 1.03Å, respectively, which indicate that all compounds strongly bind with HKII throughout the simulation.

**Figure 7:**
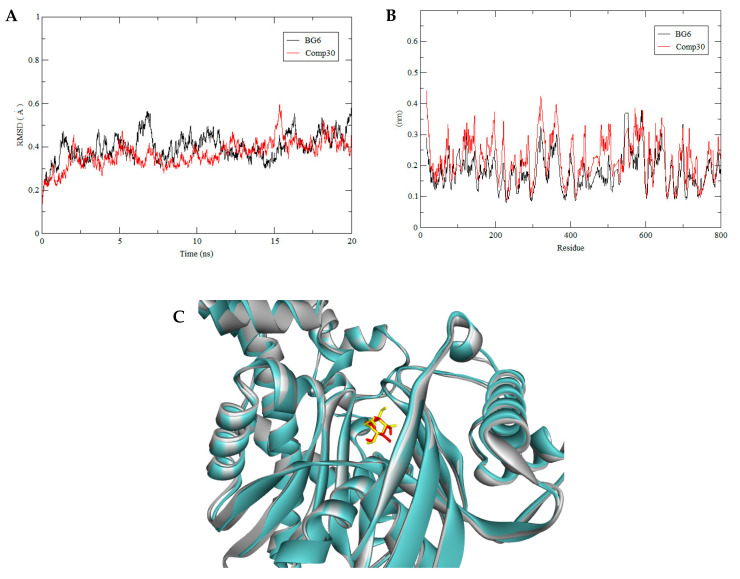
(**A**) The backbone RMSD of HKII-BG6 (black) and HKII-compound 30 (red) complexes shown during 20 ns of MD simulation. (**B**) Residue RMSF of HKII-BG6 and HKII-compound 30 complexes generated during the trajectory period of 20 ns of MD simulation. (**C**) Superimpose of trajectory structure at 10 ns and 20 ns of MD simulations. The protein was presented in a cyan-coloured ribbon format. Snapshots of superimposed ligand BG6 (red) and compound 30 (yellow) located in a ligand binding site.

**Figure 8:**
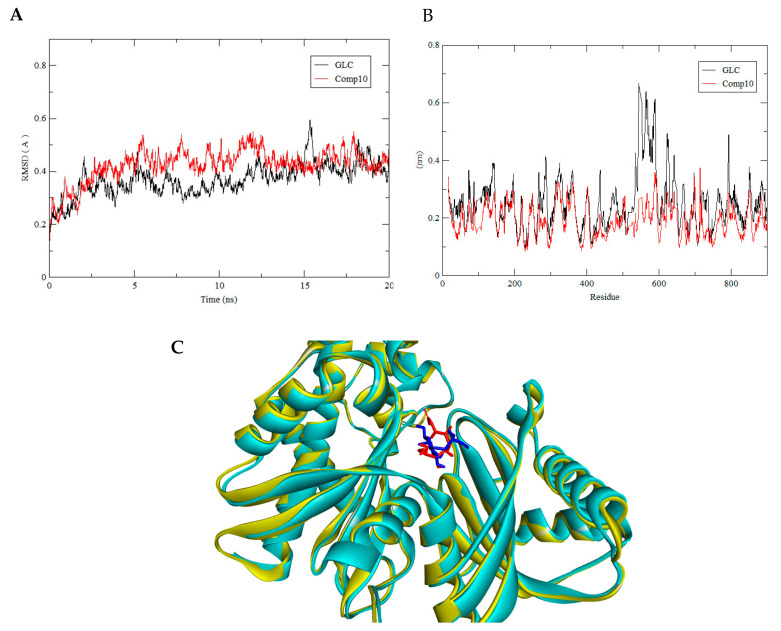
(**A**) The backbone RMSD of HKII-GLC (black) and HKII-compound 10 (red) complexes shown during 20 ns of MD simulation. (**B**) Residue RMSF of HKII-GLC and HKII-compound 10 complexes generated during the trajectory period of 20 ns of the MD simulation. (**C**) Superimpose of trajectory structure at 10 ns and 20 ns of MD simulations. The protein was presented in a cyan-coloured ribbon format. Snapshots of superimposed ligand GLC (red) and compound 10 (blue) located in a ligand binding site.

For the HKII complex with BG6 and compound 30, both were stabilized over the simulation process while the complex HKII-GLC and HKII-compound 10 were less deviated after 12 ns. Clearly, the structural dynamics of all complexes achieved stability before 20 ns. For compound 30, the backbone of the HKII complex showed comparable deviations with the reference complex, HKII-BG6, where their RMSD values were plateaued at ±4.0Å (Figure 7A), indicating that both complexes were equilibrated and achieved a favourable binding conformation with each substrate. Meanwhile, for compound 10, the backbone of HKII-compound 10 showed a similar fluctuation with the HKII-GLC with an RMSD value of ±5.0 A° (Figure 8A), indicating that both complexes behaved similarly and performed stable interactions in the aqueous environment.

All complexes (HKII-compound 30 and BG6, HKII-compound 10 and GLC) were snapshotted at 20 ns of MD production and superimposed with their reference structure to assess the binding location, as shown in Figure 7C and Figure 8C. From the superposed image, all substrates were in a close orientation with a reference and predicted to effectively inhibit HKII through a strong binding interaction. For RMSF, each amino acid fluctuation in HKII for compound 30 and compound 10 were in a similar pattern with their references BG6 and GLC, respectively. No significant changes were seen for the movement of the amino acids and the substrates were not disrupting the structural integrity of HKII.

**Figure 9:**
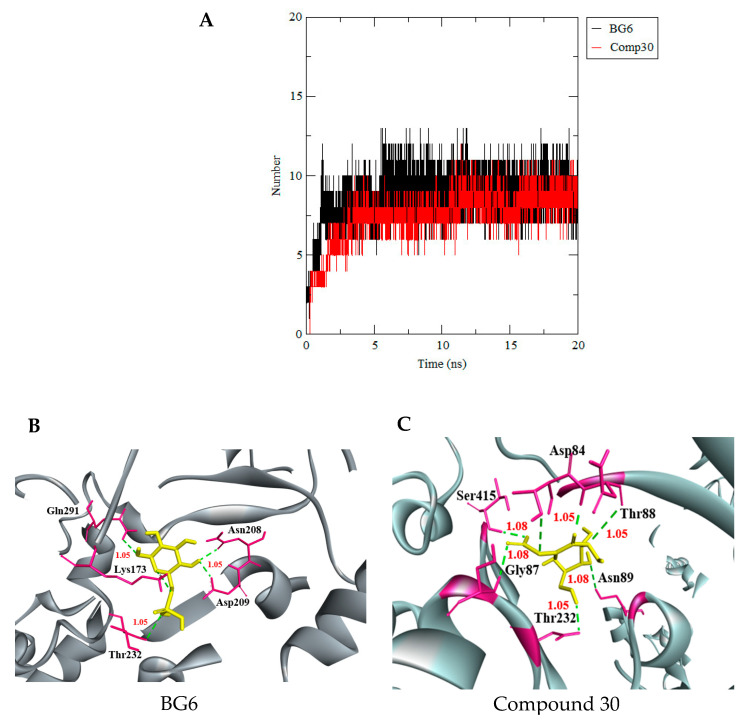
(**A**) Total number of hydrogen bond interactions between HKII and BG6 (red), and HKII and compound 30 (black). Snapshots of complex (**B**) HKII-BG6, and (**C**) HKII-compound 30 were extracted from the trajectory at 20 ns. HKII-BG6 has shown five H bonds. Rwo formed with Asn208 and Asp209, and three formed with Gln291, Lys173, and Thr232. HKII-compound 30 has shown seven H bonds. Three formed with Asp84, Asn89, and Thr88. Four formed with Ser415, Gly87, and Thr232. The distance between HKII and BG6 (yellow), and HKII and compound 30 (yellow) have an average of 1.05Å.

The binding analysis of the HKII-compound 30 revealed a higher average number of hydrogen bond (7 hydrogen bonds) per time frame during the MD simulation period (Figure 9A) compared to a hydrogen bond of the HKII-BG6 complex (five hydrogen bonds). The extracted trajectory of complex HKII-compound 30 has shown seven H bonds with Asp84, Thr88, Asn89, Thr232, Ser415, and Gly87 at 20 ns during the MD simulation, while the snapshot of complex HKII-BG6 has shown five H bonds formed with residues Asn208, Asp209, Lys173, and Gln291 at 20 ns of the MD simulation (Figure 9B,C). This analysis has suggested that compound 30 has strong interactions with HKII in the dynamic condition during MD simulation, compared to reference molecule BG6. However, compound 30 and BG6 were consistently bound in the active site of HKII and stably projected the active site vicinity. The distance between compound 30 and BG6 with HKII were below 1.05Å, which defines that their binding was strong during the simulation time with distance values mentioned in Figure 9B,C.

**Figure 10:**
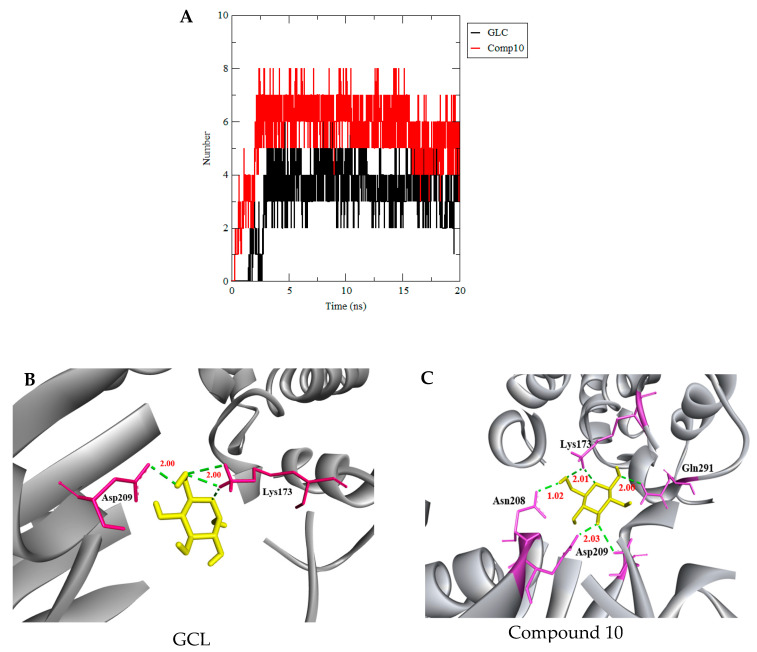
(**A**) The total number of hydrogen bond interactions between HKII and GLC (red), and HKII and compound 10 (black). Snapshots of complex (**B**) HKII-GLC and (**C**) HKII-compound10 were extracted from the trajectory at 20 ns. HKII-GLC has shown three H bonds. Two formed with Lys173 and one formed with Asp209. HKII-compound 10 has shown six H bonds. Four formed with Lys173 and Asp209 and two formed with Gln291 and Asn208. The distance between HKII and GLC (yellow), and HKII and compound 10 (yellow) are, on average, 2.00Å.

Furthermore, compound 10 with HKII has formed an average of six hydrogen bonds, which is higher than the reference complex HKII-GLC (4 hydrogen bonds) (Figure 10A). Figure 10B,C have shown the extracted trajectory of HKII-GLC and HKII-compound 10 at 20 ns, where GLC binds with HKII by four H bonds with catalytic residues Asp209 and Lys173, while compound 10 has shown six H bonds with a catalytic residue of Asn209, Lys173, Gln291, and Asp209 (Figure 10B,C). Thus, it can be said that compound 10 has more intermolecular interactions via hydrogen bonding and the compound was stably-bound in the catalytic region of HKII, showing a high level of inhibition compared to the reference molecule GLC. 

The distance between HKII and GLC as well as compound 10 were, on average, 2.0Å, which defined that their binding was strong throughout the simulation period. Several studies have reported the discovery of dengue inhibitors against the viral proteins through the MD simulation process. Shanmugam and Gromiha (2016) have conducted *insilico* processes such as molecular docking to find potential inhibitors against RNA-dependent RNA polymerase, which is essential for dengue viral replication. The researchers obtained quercetin derivatives, quercetin 3-(6″-(E)-pcoumaroylsophoroside)-7-rhamnoside as a dengue polymerase inhibitor, where this finding was validated based on the stability of the enzyme and inhibitor by an MD simulation [59]. In another study, Shanmugam and colleagues also identified, [2-(4-carbamoylpiperidin-1-yl)-2-oxoethyl] 8-(1,3-benzothiazol-2-yl) naphthalene-1-carboxylate as a potent dengue viral polymerase inhibitor by maintaining the same simulation condition from the previous study [60]. Most of the inhibitors were identified through an MD simulation against viral replication proteins, such as non-structural protein 3 (NS3), NS5 methyltransferase, and also envelope protein (E) [58,59,60,61,62,63,64]. Currently, 18 million compounds were virtually-screened against dengue replication protein NS3, where the top five compounds have shown strong predicted binding affinity for the important catalytic residues of NS3 [65]. Eventually, the top five compounds further went through the MD simulation to scrutinize the stability of selected compounds and NS3 protein throughout the simulation period [65]. In another study, 300 commercial cyclic peptides were screened against NS5 methyltransferase where two ligands, namely preproendothelin and urotensin II, were selected as the best inhibitors based on ligand-enzyme binding free energy and pharmacological prediction. Furthermore, it was confirmed that both ligands maintained a stable complex conformation with the enzymes throughout a 6-ns simulation period [64,66]. On the contrary, our findings were evaluated based on the stability of protein-ligand throughout the simulation period, where compound 1, compound 10, and compound 30, which are the analogues of 2DG, GLC, and BG6 respectively, have shown protein-ligand stability during the course of the simulation process. 

## 4. Conclusions

There is an urgent need to design better inhibitors against the DENV, in which human hexokinase II (HKII) was proposed as an ideal target for anti-DENV drug development. Precise and computationally-competent virtual screening can act as a potential step toward the future “on-shelf” dengue virus drugs. Consequently, we have identified a number of novel compounds by *in silico* screening of library of compounds from the USRCAT database using Auto Dock Vina docking programs. Docking results of the top three compounds for each reference molecule in our study have shown strong predicted binding affinity for the catalytically-important residues of HKII. Finally, compound 10, compound 30, and compound 1, which are the analogues of GLC, BG6, and 2DG, respectively, have exhibited strong protein-ligand stability with RMSD ±5.0A°, ±4.0A°, and ±0.5A throughout the simulation process. The effects of these compounds on the activity of HKII are currently being pursued in the subsequent structural and biochemical studies.

## Figures and Tables

**Table 1:** Similarity scores and Lipinski’s values for GLC and its analogues, as obtained from USRCAT.

Compound Number	ZINC ID	2D Structure	Similarity Score	Number of HBD/HBA	Molecular Weight (g/mol)	MlogP	Drug-Likeness
GLC		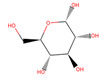		5/6	180.156	−3.21	0.01
10	3,956,760	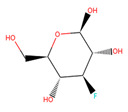	0.946	4/5	182.147	−2.15	0.14
26	16,159,409	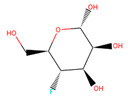	0.933	4/5	182.147	−2.15	0.09
58	3,809,846	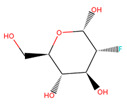	0.913	4/5	182.147	−1.93	−0.01

**Table 2:** Similarity scores and Lipinski’s values for BG6 and its analogues, as obtained from USRCAT.

Compound Number	ZINC ID	2D Structure	Similarity Score	Number of HBD/HBA	Molecular Weight (g/mol)	MlogP	Drug-Likeness
BG6		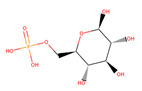		6/9	260.135	−3.64	−0.20
30	4,403,145	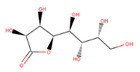	0.817	6/8	238.192	−3.84	0.89
36	4,530,268	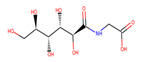	0.812	6/9	252.199	−4.02	0.72
38	1,576,959	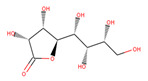	0.812	6/8	238.192	−3.84	−1.08

**Table 3:** Similarity scores and Lipinski’s values for 2DG and its analogues, as obtained from USRCAT.

Compound Number	ZINC ID	2D Structure	Similarity Score	Number of HBD/HBA	Molecular Weight (g/mol)	MlogP	Drug-Likeness
2DG		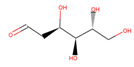		4/5	164.157	−1.95	−1.33
1	86,652,948	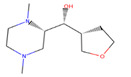	0.806	2/4	215.317	0.06	−0.64
4	86,991,606	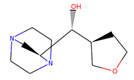	0.796	2/4	213.301	−0.08	−1.16
31	86,991,603	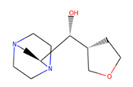	0.751	2/4	213.301	−0.08	−1.16

**Table 4:** The ADME results of GLC and analogues.

Properties	Model Name	Units	GLC	ZINC 3956760 Compound 10	ZINC16159409 Compound 26	ZINC3809846 Compound 58
Absorption	Water solubility	log mol/L	−1.377	−1.08	−1.005	−1.581
	Caco2 permeability	Log 10^−^^6^ cm/s	−0.249	0.251	0.315	0.214
	Intestinal absorption (human)	%(Absorbed)	21.51	59.553	59.79	59.316
	Skin Permeability	logKp	−3.041	−3.044	−3.107	−2.939
Distribution	Fraction unbound (human)	logL/Kg	0.82	0.841	0.84	0.853
	BBB permeability	logBB	−0.943	−0.786	−0.9	−0.872
	CNS permeability	logPS	−3.636	−3.552	−3.552	−3.192
Metabolism	CYP2D6	Yes/No	No	No	No	No
	CYP1A2	Yes/No	No	No	No	No
Excretion	Oral Rat Acute Toxicity (LD50)	mol/kg	0.626	0.535	0.535	0.534
	Renal OCT2 substrate	Yes/No	No	No	No	No

**Table 5:** The ADME results of BG6 and analogues.

Properties	Model Name	Units	BG6	ZINC 4403145 Compound 30	ZINC 4530268 Compound 36	ZINC1576959 Compound 38
Absorption	Water solubility	log mol/L	−0.811	−1.753	−1.995	−1.753
	Caco2 permeability	Log 10^−^^6^ cm/s	−0.341	−0.104	−0.371	−0.104
	Intestinal absorption (human)	%(Absorbed)	35.51	26.376	0	26.376
	Skin Permeability	logKp	−2.82	−2.744	−2.735	−2.744
Distribution	Fraction unbound (human)	logL/Kg	0.716	0.79	0.886	0.232
	BBB permeability	logBB	−1.414	−1.032	−0.928	−1.032
	CNS permeability	logPS	−4.211	−3.46	−3.604	−3.46
Metabolism	CYP2D6	Yes/No	No	No	No	No
	CYP1A2	Yes/No	No	No	No	No
Excretion	Oral Rat Acute Toxicity (LD50)	mol/kg	0.414	0.787	1.056	0.787
	Renal OCT2 substrate	Yes/No	No	No	No	No

**Table 6:** The ADME results of 2DG and analogues.

Properties	Model Name	Units	2DG	ZINC86652948 Compound 1	ZINC 86991606 Compound 4	ZINC86991603 Compound 31
Absorption	Water solubility	log mol/L	−0.512	−1.987	−1.654	−1.376
	Caco2 permeability	Log 10^−^^6^ cm/s	1.559	1.985	1.654	1.098
	Intestinal absorption (human)	%(Absorbed)	86.433	86.983	87.984	86.764
	Skin Permeability	logKp	−3.41	−3.98	−2.34	−2.75
Distribution	Fraction unbound (human)	logL/Kg	0.916	0.914	0.158	0.985
	BBB permeability	logBB	−0.043	−0.025	−0.265	−0.265
	CNS permeability	logPS	−3.434	−3.254	−3.214	−3.147
Metabolism	CYP2D6	Yes/No	No	No	No	No
	CYP1A2	Yes/No	No	No	No	No
Excretion	Oral Rat Acute Toxicity (LD50)	mol/kg	1.153	1.258	1.245	1.452
	Renal OCT2 substrate	Yes/No	No	No	No	No

**Table 7:** Toxicity values of GLC and analogues.

ZINC ID	Classification					
	Acute Toxicity	Toxicity End Point				Organ Toxicity
		Carcinogenicity	Immunotoxicity	Mutagenicity	Cytotoxicity	
GLC	**I. Toxicity Class: 6** II. LD50:2300 mg/kg III. accuracy: 70.97%	Inactive Ps: 0.82	Inactive Ps: 0.99	Inactive Ps: 0.87	Inactive Ps: 0.81	Inactive Ps: 0.98
3956760 Compound 10	**I. Toxicity Class: 6** II. LD50:14,388 mg/kg III. accuracy: 67.38%	Inactive Ps: 0.67	Inactive Ps: 0.99	Inactive Ps: 0.68	Inactive Ps: 0.68	Inactive Ps: 0.98
16159409 Compound 26	**I. Toxicity Class: 6** II. LD50:2300 mg/kg III. accuracy: 68.07%	Inactive Ps: 0.73	Inactive Ps: 0.98	Inactive Ps: 0.68	Inactive Ps: 0.64	Inactive Ps: 0.89
3809846 Compound 58	**I. Toxicity Class: 6** II. LD50:14,388 mg/kg III. accuracy: 67.38%	Inactive Ps: 0.73	Inactive Ps: 0.98	Inactive Ps: 0.68	Inactive Ps: 0.64	Inactive Ps: 0.89

**Table 8:** Toxicity values of BG6 and analogues.

ZINC ID	Classification					
	Acute Toxicity	Toxicity End Point				Organ Toxicity
		Carcinogenicity	Immunotoxicity	Mutagenicity	Cytotoxicity	
BG6	**I. Toxicity Class: 4** II. LD50:1500 mg/kg III. accuracy: 67.38%	Inactive Ps: 0.74	Inactive Ps: 0.99	Inactive Ps:0.74	Inactive Ps:0.81	Inactive Ps:0.94
4403145 Compound 30	**I. Toxicity Class: 6** II. LD50:15,900 mg/kg III. accuracy: 70.97%	Inactive Ps: 0.89	Inactive Ps: 0.99	Inactive Ps: 0.85	Inactive Ps: 0.68	Inactive Ps: 0.94
4530268 Compound 36	**I. Toxicity Class: 6** II. LD50:5500 mg/kg III. accuracy: 69.26%	Inactive Ps: 0.69	Inactive Ps: 0.99	Inactive Ps: 0.73	Inactive Ps: 0.68	Inactive Ps: 0.93
1576959 Compound 38	**I. Toxicity Class: 6** II. LD50:15,900 mg/kg III. accuracy: 70.97%	Inactive Ps: 0.89	Inactive Ps: 0.99	Inactive Ps: 0.85	Inactive Ps: 0.68	Inactive Ps: 0.94

**Table 9:** Toxicity values of 2DG and analogues.

ZINC ID	Classification					
	Acute Toxicity	Toxicity End Point				Organ Toxicity
		Carcinogenicity	Immunotoxicity	Mutagenicity	Cytotoxicity	
2DG	**I. Toxicity Class: 3** II. LD50:1500 mg/kg III. accuracy: 67.38%	Inactive Ps: 0.74	Inactive Ps: 0.99	Inactive Ps:0.74	Inactive Ps:0.81	Inactive Ps:0.94
86652948 Compound 1	**I. Toxicity Class: 6** II. LD50:15,900 mg/kg III. accuracy: 70.97%	Inactive Ps: 0.89	Inactive Ps: 0.99	Inactive Ps: 0.85	Inactive Ps: 0.68	Inactive Ps: 0.94
86991606 Compound 4	**I. Toxicity Class: 6** II. LD50:5500 mg/kg III. accuracy: 69.26%	Inactive Ps: 0.69	Inactive Ps: 0.99	Inactive Ps: 0.73	Inactive Ps: 0.68	Inactive Ps: 0.93
86991603 Compound 31	**I. Toxicity Class: 6** II. LD50:15,900 mg/kg III. accuracy: 70.97%	Inactive Ps: 0.89	Inactive Ps: 0.99	Inactive Ps: 0.85	Inactive Ps: 0.68	Inactive Ps: 0.94

**Table 10:** The top molecules, which are analogues of GLC, were ranked, according to their binding energy. The binding energy of GLC is also shown.

ZINC ID	Binding Energy (Kcal/mol) Chain A Terminal N	Hydrogen Bond Number Chain A Terminal N	Catalytic Residue
GLC	(−7.2)	6	Glu260, Lys173, Asp209, Asn235
3956760 Compound 10	(−7.2)	7	Glu260, Gln291, Phe156, Asn208, Lys173, Asp209
16159409 Compound 26	(−7.0)	5	Ser155, Asp209, Asn208, Lys173, Glu294
3809846 Compound 58	(−6.10)	2	Asp209, Asn209, Ser155

**Table 11:** The top molecules, which are analogues of BG6, were ranked according to their binding energy. The binding energy of BG6 is also shown.

ZINC ID	Binding Energy (Kcal/mol) Chain A, Terminal N	Hydrogen Bond Number Chain A Terminal N	Catalytic Residue
BG6	(−7.9)	6	Gln291, Pro157, Lys173, Thr232, Gly87, Asp209, Asn208
4403145 Compound 30	(−7.8)	11	Gly87, Asn89, Thr232, Arg91, Asp84, Lys173
4530268 Compound 36	(−7.4)	9	Asp84, Thr88, Thr323, Asn89
1576959 Compound 38	(−7.0)	5	Asn89, Thr88, Ser449

**Table 12:** The top molecules, which are analogues of 2-DG, were ranked according to their binding energy. The binding energy of 2-DG is also shown.

ZINC ID	Binding Energy (Kcal/mol) Chain A Terminal N	Hydrogen Bond Number Chain A Terminal N	Catalytic Residue
2DG	(−6.0)	8	Asn208, Asp209, Thr172, Lys173, Glu294, Glu260, Phe156
86652948 Compound 1	(−6.8)	4	Asp209, Asn208, Glu294, Thr172, Thr232, Lys173
86991606 Compound 4	(−6.3)	3	Asp209, Asp84, Gly87, Thr88, Asp413, Thr232
86991603 Compound 31	(−6.3)	1	Asp413, Asp84, Asp209

## Data Availability

The data presented in this study are available in this article “Virtual Screening for Potential Inhibitors of Human Hexoki-nase II for the Development of Anti-Dengue Therapeutics”.
